# Bounds for Coding Theory over Rings

**DOI:** 10.3390/e24101473

**Published:** 2022-10-16

**Authors:** Niklas Gassner, Marcus Greferath, Joachim Rosenthal, Violetta Weger

**Affiliations:** 1 Institute of Mathematics, University of Zurich, 8057 Zurich, Switzerland; 2 School of Mathematics and Statistics, University College of Dublin, D04 V1W8 Dublin, Ireland; 3 Department of Computer Engineering, Technical University of Munich, 80333 München, Germany

**Keywords:** rings, coding theory, Johnson bound, Plotkin bound

## Abstract

Coding theory where the alphabet is identified with the elements of a ring or a module has become an important research topic over the last 30 years. It has been well established that, with the generalization of the algebraic structure to rings, there is a need to also generalize the underlying metric beyond the usual Hamming weight used in traditional coding theory over finite fields. This paper introduces a generalization of the weight introduced by Shi, Wu and Krotov, called overweight. Additionally, this weight can be seen as a generalization of the Lee weight on the integers modulo 4 and as a generalization of Krotov’s weight over the integers modulo 2^*s*^ for any positive integer s. For this weight, we provide a number of well-known bounds, including a Singleton bound, a Plotkin bound, a sphere-packing bound and a Gilbert–Varshamov bound. In addition to the overweight, we also study a well-known metric on finite rings, namely the homogeneous metric, which also extends the Lee metric over the integers modulo 4 and is thus heavily connected to the overweight. We provide a new bound that has been missing in the literature for homogeneous metric, namely the Johnson bound. To prove this bound, we use an upper estimate on the sum of the distances of all distinct codewords that depends only on the length, the average weight and the maximum weight of a codeword. An effective such bound is not known for the overweight.

## 1. Introduction

Coding theoretic experience has shown that considering linear codes over finite fields often yields significant complexity advantages over the nonlinear counterparts, particularly when it comes to complex tasks such as encoding and decoding. On the other side, it was recognized early [1,2] that the class of binary block codes contains excellent code families, which were not linear (Preparata, Kerdock codes, Goethals and Goethals–Delsarte codes). For a long time, it could not be explained why these families exhibit formal duality properties in terms of their distance enumerators that occur only on those among linear codes and their duals.

A true breakthrough in the understanding of this behavior came in the early 1990s when, after preceding work by Nechaev [3], the paper by Hammons et al. [4] discovered that these families allow a representation in terms of $Z_{4}$-linear codes.

A crucial condition for this ring-theoretic representation was that $Z_{4}$ was equipped with an alternative metric, the Lee weight, rather than with the traditional Hamming weight, which only distinguishes whether an element is zero or non-zero. The Lee weight is finer, assigning 2 a higher weight than the other non-zero elements of this ring.

The fact that the traditional settings of linear coding theory (finite fields endowed with the Hamming metric) are actually too narrow, which suggests expanding the theory in at least two directions: on the algebraic part, the next more natural algebraic structure serving as alphabet for linear coding is that of finite rings (and modules). On the metrical part, the appropriateness of the Lee weight for $Z_{4}$-linear coding suggests that the distance function for a generalized coding theory requires generalization as well.

Since these ground-breaking observations, an entire discipline arose within algebraic coding theory. A considerable community of scholars have been developing results in various directions, among them code duality, weight-enumeration, code equivalence, weight functions, homogeneous weights, existence bounds, code optimality and decoding schemes, to mention only a few.

The paper at hand aims at providing a further contribution to this discipline, by introducing the overweight on a finite ring. This weight is a generalization of the Lee weight over $Z_{4}$, as well as of the weight introduced in [5] by Krotov over $Z_{2^{s}}$ for any positive integer *s*, which was further generalized to $Z_{p^{k}}$ in [6].

We study the relations of this new weight to other well-known weights over rings and state several properties of the overweight, such as its extremal property. We also develop a number of standard existence bounds, such as a Singleton bound, a sphere-packing bound, a Plotkin bound and a version of the (assertive) Gilbert–Varshamov bound.

In the final part of this article, we derive a general Johnson bound for the homogeneous weight on a finite Frobenius ring. This result is important, as it is closely connected to list decoding capabilities.

## 2. Preliminaries

Throughout this paper, we will consider *R* to be a finite ring with identity, denoted by 1. If *R* is a finite ring, we denote by $R^{\times }$ its group of invertible elements, also known as units.

Let us recall some preliminaries in coding theory, where we focus on ring-linear coding theory.

For a prime power *q*, let us denote by $F_{q}$ the finite field with *q* elements and, for a positive integer *m*, we denote by $Z_{m}$ the ring of integers modulo *m*.

In traditional coding theory, we consider a linear code to be a subspace of a vector space over a finite field.

**Definition** **1.** *Let q be a prime power, and let k≤n be non-negative integers. A linear subspace C of $F_{q}^{n}$ of dimension k is called a linear [n,k] code.*

We define a weight in a general way.

**Definition** **2.** *Let R be a finite ring. A real-valued function w on R is called a* weight *if it is a non-negative function that maps 0 to 0.*

It is natural to identify *w* with its additive extension to $R^{n}$, and so we will always write $w\left(x\right)=\sum_{i=1}^{n}w\left(x_{i}\right)$ for all $x\in R^{n}$. Every weight *w* on *R* induces a *distance* d:R×R⟶R by d(x,y)=w(x−y). Again, we will identify *d* with its natural additive extension to $R^{n}\times R^{n}$.

If the weight additionally is positive definite, symmetric and satisfies the triangular inequality, that is,

1. w(0)=0 and w(x)>0 for all x≠0,
2. w(x)=w(−x) for all x∈R,
3. w(x+y)≤w(x)+w(y) for all x,y∈R,

then the induced distance inherits these properties, i.e.,

1. d(x,y)≥0 for all x,y∈R and d(x,y)=0 if and only if x=y.
2. d(x,y)=d(y,x) for all x,y∈R,
3. d(x,z)≤d(x,y)+d(y,z) for all x,y,z∈R.

The most prominent and best studied weight in traditional coding theory is the Hamming weight.

**Definition** **3.** *Let n∈N. The Hamming weight of a vector $x\in R^{n}$ is defined as the size of its support*

$$w_{H}\left(x\right)=\left|\{i\in \left\{1,\ldots ,n\right\}|x_{i}\neq 0\}\right|,$$

*and the Hamming distance between x and $y\in R^{n}$ is given by*

$$d_{H}\left(x,y\right)=\left|\{i\in \left\{1,\ldots ,n\right\}|x_{i}\neq y_{i}\}\right|=w_{H}\left(x-y\right).$$

The minimum Hamming distance of a code is then defined as the minimum distance between two different codewords

$$d_{H}\left(C\right)=\min\left\{d_{H}\left(x,y\right)|x,y\in C,\;x\neq y\right\}.$$

Note that the concept of minimum distance can be applied for any underlying distance *d*.

In the paper at hand, we focus on a more general setting where the ambient space is a module over a finite ring.

**Definition** **4.** *Let n∈N and let R be a finite ring. A submodule C of ${}_{R}R^{n}$ of size M=|C| is called a left R-linear (n,M) code.*

The most studied ambient space for ring-linear coding theory is the integers modulo 4, denoted by $Z_{4}$, endowed with the Lee metric.

**Definition** **5.** *For $x\in Z_{m}$, its Lee weight is defined as*

$$w_{L}\left(x\right)=\min\left\{x,|m-x|\right\}.$$

One of the most prominent generalizations of the Lee weight over $Z_{4}$ is the homogeneous weight.

**Definition** **6.**  *Let R be a Frobenius ring. A weight w:R⟶R is called (left) homogeneous of average value γ>0, if w(0)=0 and the following conditions hold:*

*(i)*  *For all x,y with Rx=Ry, we have that w(x)=w(y).*
*(ii)*  *For every non-zero ideal $I\leq {}_{R}R$, it holds that*
$$\frac{1}{\left|I\right|}\sum_{x\in I}w\left(x\right)\;=\;\gamma .$$

*We will denote the homogeneous weight with wt.*

The homogeneous weight was first introduced by Constantinescu and Heise in [7] in the context of coding over integer residue rings. It was later generalized by Greferath and Schmidt [8] to arbitrary finite rings, where the ideal *I* in Definition 6 was assumed to be a principal ideal. In its original form, however, the homogeneous weight only exists on finite Frobenius rings. It can be shown that a left homogeneous weight is at the same time right homogeneous, and for this reason, we will omit the reference to any side for the sequel. In [9], Honold and Nechaev finally generalized the notion of homogeneous weight to some finite modules, called weighted modules, over a (not necessarily commutative) ring *R* with identity.

Since we will establish a Plotkin bound for a new weight, let us recall here the Plotkin bound over finite fields equipped with the Hamming metric.

**Theorem** **1** (Plotkin bound). *Let C be an (n,M) block code over $F_{q}$ with minimum Hamming distance d. If $d>\frac{q-1}{q}n$, then*

$$M\;\leq \;\frac{d}{d-\frac{q-1}{q}n}.$$

For the homogeneous weight, the following Plotkin bound was established in [10].

**Theorem** **2** (Plotkin bound for homogeneous weights, [10]). *Let wt be a homogeneous weight of average value γ on R, and let C be an (n,M) block code over R with minimum homogeneous distance d. If γn<d, then*

$$M\;\leq \;\frac{d}{d-\gamma n}.$$

## 3. Overweight

As the Hamming weight defined over the binary can be generalized to larger ambient spaces in different ways resulting in different metrics, such as the Hamming weight over $F_{q}$ or the Lee weight over $Z_{p^{s}}$; in addition, the Lee weight over $Z_{4}$ can be generalized in different ways. For example, the weight defined in [5] over $Z_{2m}$ for any positive integer *m* is a possible generalization, but the most prominent generalization is the homogeneous weight (see for example [10]). In this section, we introduce a new generalization, called the *overweight*. This weight shows some interesting properties and relations to the homogeneous weight and can additionally be seen as a generalization of the weight defined in [5] over $Z_{2^{s}}$ for any positive integer *s* and the weight defined in [6] over $Z_{p^{s}}$.

**Definition** **7.**  *Let R be a finite ring. The* overweight *on R is defined as*

$$W:R⟶R,\;x\mapsto \left\{\begin{array}{cc} 0 & \mathrm{if}\;x=0,\\ 1 & \mathrm{if}\;x\in R^{\times },\\ 2 & \mathrm{otherwise}. \end{array}\right.$$

We also denote by *W* its additive expansion to $R^{n}$, given by $W\left(x\right)=\sum_{i=1}^{n}W\left(x_{i}\right).$

Let us call the distance which is induced by the overweight the *overweight distance*, and denote it by *D*, i.e., D(x,y)=W(x−y).

The motivation of introducing this new weight is twofold: on one hand, it is theoretically interesting to explore a new generalization of the Lee weight over $Z_{4}$ and its connections to other known weights over rings. On the other hand, the overweight would also be perfectly suitable for a channel, where unit errors are more likely.

Note that the overweight is designed to satisfy the following criteria: it is positive definite, symmetric, satisfies the triangular inequality and distinguishes between units and non-zero non-units. Furthermore, it is extremal in the sense that, on a big family of rings, any increase of the weight of non-zero non-units would violate the triangular inequality, thus the name *overweight*. We will now study this extremal property in more details.

We can consider weights with values in {0,1,α}, for some α>0, without fixing the subsets of *R* where these values are attained. Thus, we are considering the generic weight function

$$\begin{array}{c} f\left(x\right)=\left\{\begin{array}{cc} 0 & \mathrm{if}\;x=0,\\ 1 & \mathrm{if}\;x\in A_{1},\\ \alpha & \mathrm{if}\;x\in A_{2}, \end{array}\right. \end{array}$$

where $A_{1}\subset R\backslash \left\{0\right\}$ and $A_{2}=R\backslash \left(A_{1}\cup \left\{0\right\}\right).$ Such a weight is always positive definite. In addition, the weight is symmetric if and only if $A_{1}$ and $A_{2}$ contain all additive inverses of their elements. Let us now consider the triangular inequality: if there exist $x,y\in A_{1}$ such that $x+y\in A_{2}$, then we must have

α=f(x+y)≤f(x)+f(y)=2.

Thus, in order for *f* to be an extremal weight, one chooses α=2.

The overweight is a special case of such a weight function *f* with the choice $A_{1}=R^{\times }$. The existence of elements $x,y\in R^{\times }$ such that $x+y\in R\backslash (\left\{0\right\}\cup R^{\times })$ is satisfied for many rings—for example, for rings with a non-trivial Jacobson radical.

### Relations to Other Weights

Clearly, the homogeneous weight and the overweight coincide with the Lee weight on $Z_{4}$, with the Hamming metric on finite fields $F_{q}$, and finally with the weight [6] on $Z_{p^{s}}.$

**Proposition** **1.** *The overweight over finite chain rings gives an upper bound on the normalized homogeneous weight.*

**Proof.**  Over a finite chain ring with socle *S* and residue field size *q*, we have that the normalized homogeneous weight is defined as

$$\begin{array}{c} wt\left(x\right)=\left\{\begin{array}{cc} 0 & \mathrm{if}\;x=0,\\ \frac{q}{q-1} & \mathrm{if}\;x\in S\backslash \{0\},\\ 1 & \mathrm{else}. \end{array}\right. \end{array}$$

If x∈S\{0}, then also $x\in R\backslash R^{\times }$, and

$$wt\left(x\right)=\frac{q}{q-1}\leq 2=W\left(x\right).$$

If $x\in R^{\times }$, then wt(x)=1=W(x) and finally, if $x\in R\backslash (S\cup R^{\times })$, we have that

wt(x)=1≤2=W(x),

which implies the result. □

In [11], Bachoc defines the following weight on $F_{p}$-algebras *A*, with units $A^{\times }$ as follows:

$$\begin{array}{c} w_{B}\left(x\right)=\left\{\begin{array}{cc} 0 & \mathrm{if}\;x=0,\\ 1 & \mathrm{if}\;x\in A^{\times },\\ p & \mathrm{else}. \end{array}\right. \end{array}$$

This is in the same spirit as the overweight. The weight of Bachoc is, however, only assuming positive definiteness. We note that, whenever we have a $F_{2}$-algebra, the two weights coincide. The overweight can thus also be seen as a generalization of Bachoc’s weight to a general finite ring.

Let us illustrate this connection with some examples: we consider the ring $M_{2}\left(F_{p}\right)$ of 2×2 matrices over $F_{p}$ and the ring $F_{p}\left[x\right]/\left(x^{2}\right)$. In both cases, the Bachoc weight only coincides with the homogeneous and the overweight in the case p=2.

Finally, in [5], Krotov defines the following weight over $Z_{2m}$, for any positive integer *m*:

$$\begin{array}{c} w_{K}\left(x\right)=\left\{\begin{array}{cc} 0 & \mathrm{if}\;x=0,\\ 2 & \mathrm{if}\;2|x,x\neq 0,\\ 1 & \mathrm{else}. \end{array}\right. \end{array}$$

Clearly, this is a further generalization of the Lee weight over $Z_{4}$ and thus coincides there with the homogeneous and the overweight. However, even more is true: the weight of Krotov and the overweight coincide over $Z_{2^{s}}$, for any positive integer *s*. Thus, the overweight may be considered as a generalization of Krotov’s weight over $Z_{2^{s}}$ for any positive integer *s*.

Let us give some examples to illustrate the differences between the above-mentioned weights.

**Example** **1.** *In the following table, $w_{H}$ denotes the Hamming weight, wt the normalized homogeneous weight, $w_{L}$ denotes the Lee weight, $w_{K}$ denotes Krotov’s weight, $w_{B}$ denotes Bachoc’s weight and finally W denotes the overweight. Let us consider two easy but pathological cases, namely $Z_{6}$ for Table 1 and $Z_{2}\times Z_{2}$ for Table 2.*

Finally, another interesting connection to the Hamming weight arises by considering the following linear injective isometry.

**Lemma** **1.** *The map*

$$\begin{array}{cc} \psi :(F_{2}\left[x\right]/\left(x^{2}\right),W) & \to (F_{2}^{2},w_{H})\\ a+bx & \mapsto (a+b,b) \end{array}$$

*is a linear isometry.*

Recall that, over $F_{2}\left[x\right]/\left(x^{2}\right)$, the overweight coincides with the weight of Bachoc and the homogeneous weight.

## 4. Bounds for the Overweight

In this section, we develop several bounds for the overweight, such as a Singleton bound, a sphere-packing bound, a Gilbert–Varshamov bound and a Plotkin bound.

For this, let us first define the minimum overweight distance of a code.

**Definition** **8.** *Let $C\subseteq R^{n}$ be a code. The minimum overweight distance of C is then denoted by D(C) and defined as*

D(C)=min{D(x,y)∣x,y∈C,x≠y}.

### 4.1. A Singleton Bound

The Singleton bound usually follows a puncturing argument, which is possible for the overweight, but gives the same result as applying the following observation:

**Remark** **1.** *For all x∈R, we have that*

$$0\leq w_{H}\left(x\right)\leq W\left(x\right)\leq 2w_{H}\left(x\right)\leq 2n,$$

*where $w_{H}$ denotes the Hamming weight.*

Hence, using the Singleton bound for the Hamming metric directly gives a Singleton bound for the overweight.

**Proposition** **2.** *Let $C\subseteq R^{n}$ be a code of size M and minimum overweight distance d. Then,*

$$d\leq 2(n-\left\lceil \log_{|R|}\left(M\right)\right\rceil +1).$$

**Example** **2.** *A trivial example for a code achieving the Singleton bound in Proposition 2 is given by $C=\langle \left(p,\ldots ,p\right)\rangle \subset Z_{p^{s}}^{n}$, having $\log_{p^{s}}\left(|C|\right)=\frac{s-1}{s}$ and minimum overweight distance d=2n.*

However, if we define the rank of a linear code *C*, denoted by rk(C), to be the minimal number of generators of *C*, then the following bound is known for principal ideal rings [12,13]

$$d_{H}\left(C\right)\leq n-rk\left(C\right)+1.$$

Codes achieving this bound are called Maximum Distance with respect to Rank (MDR) codes, in order to differentiate from MDS codes. This is a sharper bound than the usual Singleton bound, since for non-free codes we have $rk\left(C\right)>\log_{|R|}\left(M\right)$.

In the case of linear codes, the rank thus also leads to a sharper Singleton-like bound for the overweight.

**Proposition** **3.** *Let R be a principal ideal ring. Let $C\subseteq R^{n}$ be a linear code of rank rk(C) and minimum overweight distance d. Then,*

d≤2(n−rk(C)+1).

**Example** **3.** *As an example for a code, we can consider $C=\langle \left(3,6,3,0\right),\left(6,6,0,3\right)\rangle \subset Z_{9}^{4}$, having minimum overweight distance d=6.*

### 4.2. A Sphere-Packing Bound

The sphere-packing bound as well as the Gilbert–Varshamov bound are *generic* bounds, and we are able to provide them for the overweight in a simple form involving the volume of the balls in the underlying metric space.

We begin by defining balls with respect to the overweight distance.

**Definition** **9.** *For a given radius r≥0, the* overweight ball *$B_{r,D}\left(x\right)$ of radius r centered in x is defined as*

$$B_{r,D}\left(x\right)\;:=\;\left\{y\in R^{n}|D\left(x,y\right)\leq r\right\}.$$

Clearly, the volume of such a ball is invariant under translations, i.e.,

$$\left|B_{r,D}\left(x\right)\right|\;=\;\left|B_{r,D}\left(y\right)\right|,$$

for all $x,y\in R^{n}$.

Moreover, setting $u:=|R^{\times }|$ and v:=|R|−1−u, we have the generating function $f_{W}\left(z\right)=1+uz+vz^{2}$ for this weight function, so that the generating function for *W* on $R^{n}$ takes the form

fWn(z)=(1+uz+vz2)n=∑k0+ku+kv=nnk0,ku,kv1k0(uz)ku(vz2)kv=∑k=0n∑ℓ=0n−knkn−kℓukvℓzk+2ℓ,

where we have set $k=k_{u}$ and $\ell =k_{v}$, and where the condition $k_{0}+k_{u}+k_{v}=n$ is transformed in 0≤k≤n, 0≤ℓ≤n−k. Now, setting t=k+2ℓ, we obtain the simplified expression for the generating function

fWn(z)=∑t=02n∑ℓ=0⌊t2⌋nt−2ℓn−t+2ℓℓut−2ℓvℓzt.

**Lemma** **2.** *The foregoing implies that the ball of radius e (centered in 0) has volume exactly*

(1) Be,D(0)=∑t=0e∑ℓ=0⌊t2⌋nt−2ℓn−t+2ℓℓut−2ℓvℓ.

We thus provided an explicit formula for the cardinality of balls in $R^{n}$ with respect to the overweight distance.

We now obtain the sphere-packing bound for the overweight distance by combining the previous results. As before, *R* is a finite ring and $u=|R^{\times }|$, whereas v=|R|−1−u represents the number of non-zero non-units.

**Corollary** **1** (Sphere-Packing Bound). *Let $C\subseteq R^{n}$ be a (not necessarily linear) code of length n, and minimum overweight distance d=2e+1. Then, we have*

$$|C|\;\leq \;\frac{{\left|R\right|}^{n}}{\left|B_{e,D}\left(0\right)\right|},$$

*where the cardinality of $B_{e,D}\left(0\right)$ is given in Equation* (1).

If the minimum distance is even and *R* is a finite local ring with maximal ideal *J*, this bound can be adapted as follows.

**Corollary** **2.** *Let R be a local ring with maximal ideal J, q=|R/J| and $C\subseteq R^{n+1}$ be a (not necessarily linear) code of length n+1 and minimum overweight distance d=2e+2. Then,*

$$|C|\;\leq \;\frac{{\left|R\right|}^{n+1}}{q\left|B_{e,D}\left(0\right)\right|},$$

*where $B_{e,D}\left(0\right)$ is the overweight ball of radius e in $R^{n}$, and its volume is given in Equation* (1).

**Proof.**  Pick $x_{1},\ldots ,x_{q}$ such that the cosets $x_{1}+J,\ldots ,x_{q}+J$ form a partition of *R*. For all m∈J, define the set

$$S_{m}:=\left\{x_{1}+m,\ldots ,x_{q}+m\right\}.$$

Notice that the sets $S_{m}$ form a partition of *R* and that all elements of $S_{m}$ have mutual overweight distance 1. Thus, given r∈R, we denote with S(r) the unique set $S_{m}$ that contains *r*. Furthermore, let

$$\pi :R^{n+1}\to R^{n}$$

be the projection that removes the n+1’th coordinate and

$$Z\left(x\right):=\{z\in R^{n+1}\;|\;D\left(\pi \left(z\right),\pi \left(x\right)\right)\leq e,\;z_{n+1}\in S\left(x_{n+1}\right)\}.$$

Now, if $x\neq y\in R^{n+1}$ are two codewords, then Z(x) and Z(y) are disjoint. Indeed, if z∈Z(x)∩Z(y), then $S\left(x_{n+1}\right)=S\left(y_{n+1}\right)$ as they cannot be disjoint. Hence, $D(x_{n+1},y_{n+1})\leq 1$. Furthermore, both D(π(x),π(z)) and D(π(y),π(z)) are less than or equal to *e*, implying that D(π(x),π(y))≤2e. It follows that D(x,y)≤2e+1, which is a contradiction. □

To find non-trivial examples of perfect codes is as notoriously hard as over finite fields in the Hamming metric. Clearly, in the case $R=F_{q}$, there are non-trivial perfect codes, as the overweight coincides with the Hamming weight. Examples of such codes can be found in [5] (Section IV). Furthermore, in the case $R=Z_{p^{k}}$, linear 1-perfect codes are classified in terms of their parity-check matrix in [6] (Theorem IV.1).

### 4.3. A Gilbert–Varshamov Bound

With arguments similar to those for the sphere-packing bound, we can also obtain a lower bound on the maximal size of a code with a fixed minimum distance.

**Proposition** **4** (Gilbert–Varshamov bound). *Let R be a finite ring, n a positive integer and d∈{0,…,2n}. Then, there exists a code $C\subseteq R^{n}$ of minimum overweight distance at least d satisfying*

$$|C|\;\geq \;\frac{{\left|R\right|}^{n}}{\left|B_{d-1,D}\left(0\right)\right|},$$

*where the volume is given in Equation* (1) *for e=d−1, i.e.,*

Bd−1,D(0)=∑t=0d−1∑ℓ=0⌊t2⌋nt−2ℓn−t+2ℓℓut−2ℓvℓ.

**Proof.**  Assume $C\subseteq R^{n}$ of minimum overweight distance of at least *d* is a largest code of length *n* and minimum distance *d*. Then, the set of balls $B_{d-1,D}\left(x\right)$ centered in the codewords x∈C must already cover the space $R^{n}$. Since, if this were not the case, one would find an element $y\in R^{n}$ that is not contained in the ball of radius d−1 around any element of *C*. This word *y* would have distance at least *d* to each of the words of *C*, and thus C∪{y} would be a code of properly larger size with distance at least *d*, a contradiction to the choice of *C*.

From the covering argument, we then see that

$$|C|\;\geq \;\frac{{\left|R\right|}^{n}}{\left|B_{d-1,D}\left(0\right)\right|},$$

as desired. □

Let us consider the special case where *R* is a finite chain ring. Since the overweight is an additive weight, and the conditions of [14] are easily verified, we can use [14] (Theorem 22) to obtain that random linear codes over $R^{n}$ achieve the (asymptotic) Gilbert–Varshamov bound with high probability.

**Example** **4.** *As an easy example, we can consider $R^{n}=Z_{8}^{2}$. The maximal minimum overweight distance is given by d=2n=4. The Gilbert–Varshamov bound states for this example that there exists a code C with ∣C∣>1, as $|B_{3,D}\left(0\right)|=55$. For example, the code C=〈(2,2)〉 has four elements.*

### 4.4. A Plotkin Bound

Over a local ring, we can use methods similar to the ones used for the classical Plotkin bound to obtain an analogue of the Plotkin bound for (not necessarily linear) codes equipped with the overweight.

For the rest of this section, *R* is a finite local ring with maximal ideal *J*. The notation stems from the Jacobson radical of the ring *R*. Note that the factor ring R/J is a finite field, whose cardinality will be denoted by *q*.

Similarly to the Hamming case, for a subset A⊆R, we will denote by

$$\bar{W}\left(A\right)=\frac{\sum_{a\in A}W\left(a\right)}{\left|A\right|}$$

the average weight of the subset *A*.

**Lemma** **3.** *Let I⊆R be a left or right ideal. Then,*

$$\bar{W}\left(I\right)=\left\{\begin{array}{cc} \frac{|R|+|J|-2}{\left|R\right|} & \mathrm{if}\;I=R,\\ 2\left(1-\frac{1}{\left|I\right|}\right) & \mathrm{if}\;\{0\}⊊I⊊R,\\ 0 & \mathrm{else}. \end{array}\right.$$

**Proof.**  Note that the last case is trivial as I={0}. If {0}⊊I⊊R, then all non-zero elements of *I* have weight 2, so this case follows as well.

Finally, if I=R, then there are |R\J|=|R|−|J| elements of weight 1 and |J|−1 elements of weight 2. Hence, the total weight is |R|−|J|+2(|J|−1) and dividing by |R| yields the claim. □

**Corollary** **3.** *Let R be a local ring with maximal ideal J and assume that |J|≥2. Then, we have that $\bar{W}\left(J\right)\geq \bar{W}\left(I\right)$ for all left or right ideals I⊆R.*

**Proof.**  We immediately see that $\bar{W}\left(J\right)\geq \bar{W}\left(I\right)$ for all I⊆J. Now, consider the case I=R. We have that

$$\begin{array}{cc} \bar{W}\left(R\right) & =\frac{|R|+|J|-2}{\left|R\right|}=\frac{\left|R\backslash J\right|}{\left|R\right|}+2\frac{|J|-1}{\left|R\right|}\\ \; & =\frac{\left|R\backslash J\right|}{\left|R\right|}+2\frac{|J|-1}{\left|J\right|}\cdot \frac{\left|J\right|}{\left|R\right|}\\ \; & \leq 2\frac{|J|-1}{\left|J\right|}\cdot \frac{\left|R\backslash J\right|}{\left|R\right|}+2\frac{|J|-1}{\left|J\right|}\cdot \frac{\left|J\right|}{\left|R\right|}\\ \; & =2\frac{|J|-1}{\left|J\right|}=\bar{W}\left(J\right), \end{array}$$

where we used that $2\frac{|J|-1}{\left|J\right|}\geq 1$. □

To ease the notation, let us denote by η the following

$$\eta =\bar{W}\left(J\right)=2\left(1-\frac{1}{\left|J\right|}\right).$$

In what follows, we provide a Plotkin bound for the overweight over a local ring *R* with maximal ideal *J*. The case |J|=1 is already well studied, since, in this case, *R* is a field and *D* is simply the Hamming distance. Hence, we will assume that |J|≥2.

We start with a lemma for the Hamming weight. The proof of it follows the idea of the classical Plotkin bound, which can be found in [15], and for the homogeneous weight in [10].

**Lemma** **4.** *Let I⊆R be a subset and P be a probability distribution on I. Then, we have that*

$$\sum_{x\in I}\sum_{y\in I}w_{H}\left(x-y\right)P\left(x\right)P\left(y\right)\leq 1-\frac{1}{\left|I\right|}.$$

**Proof.**  We have that

$$\begin{array}{c} \sum_{x\in I}\sum_{y\in I}w_{H}\left(x-y\right)P\left(x\right)P\left(y\right)=\sum_{x\in I}P\left(x\right)\left(1-P\left(x\right)\right)=\sum_{x\in I}P\left(x\right)-\sum_{x\in I}P{\left(x\right)}^{2}. \end{array}$$

If we apply the Cauchy–Schwarz inequality to the latter sum, we obtain that

$$\begin{array}{c} \sum_{x\in I}P\left(x\right)-\sum_{x\in I}P{\left(x\right)}^{2}\leq 1-\frac{1}{\left|I\right|}{\left|\sum_{x\in I}P\left(x\right)\right|}^{2}=1-\frac{1}{\left|I\right|}. \end{array}$$

From which we can conclude. □

We are now ready for the most important step of the Plotkin bound. As before, *R* is a local ring with non-zero maximal ideal *J* and $\eta =\bar{W}\left(J\right)$.

**Proposition** **5.** *Let P be a probability distribution on R. Then, it holds that*

$$\sum_{x\in R}\sum_{y\in R}W\left(x-y\right)P\left(x\right)P\left(y\right)\leq \eta .$$

**Proof.**  Let q=|R/J| and pick $x_{1},\ldots ,x_{q}$ such that $x_{i}+J\neq x_{j}+J$ if i≠j. Then, it follows that the cosets $\bar{x_{i}}:=x_{i}+J$ form a partition of *R*. For all k∈{1,…,q}, we denote by

$$P_{k}=\sum_{x\in \bar{x_{k}}}P\left(x\right).$$

It follows that $\sum_{k=1}^{q}P_{k}=1$. By rewriting the initial sum as sum over all cosets, we obtain that

$$\begin{array}{cc} \; & \sum_{x\in R}\sum_{y\in R}W\left(x-y\right)P\left(x\right)P\left(y\right)\\ = & \sum_{k=1}^{q}\sum_{x\in \bar{x_{k}}}\sum_{y\in R}W\left(x-y\right)P\left(x\right)P\left(y\right)\\ = & \sum_{k=1}^{q}\sum_{x\in \bar{x_{k}}}\left(\sum_{y\in \bar{x_{k}}}2w_{H}\left(x-y\right)P\left(x\right)P\left(y\right)+\sum_{z\in R\backslash \bar{x_{k}}}w_{H}\left(x-z\right)P\left(x\right)P\left(z\right)\right)\\ = & \sum_{k=1}^{q}\left(2\sum_{x\in \bar{x_{k}}}\sum_{y\in \bar{x_{k}}}w_{H}\left(x-y\right)P\left(x\right)P\left(y\right)+\sum_{x\in \bar{x_{k}}}\sum_{z\in R\backslash \bar{x_{k}}}P\left(x\right)P\left(z\right)\right)\\ = & \sum_{k=1}^{q}\left(2\sum_{x\in \bar{x_{k}}}\sum_{y\in \bar{x_{k}}}w_{H}\left(x-y\right)P\left(x\right)P\left(y\right)+\sum_{x\in \bar{x_{k}}}P\left(x\right)\left(1-P_{k}\right)\right). \end{array}$$

If $P_{k}\neq 0$, then $\tilde{P}\left(x\right):=P\left(x\right)/P_{k}$ defines a probability distribution on $\bar{x_{k}}$. In this case, we apply Lemma 4 to obtain that

$$\begin{array}{cc} \; & \sum_{x\in \bar{x_{k}}}\sum_{y\in \bar{x_{k}}}w_{H}\left(x-y\right)P\left(x\right)P\left(y\right)\\ = & P_{k}^{2}\left(\sum_{x\in \bar{x_{k}}}\sum_{y\in \bar{x_{k}}}w_{H}\left(x-y\right)\frac{P(x)P(y)}{P_{k}^{2}}\right)\\ \leq & P_{k}^{2}\left(1-\frac{1}{\left|J\right|}\right). \end{array}$$

Note that the same inequality also trivially holds if $P_{k}=0$. Applying this and using that $\sum_{x\in \bar{x_{k}}}P\left(x\right)=P_{k}$, we obtain that

$$\begin{array}{cc} \; & \sum_{k=1}^{q}\left(2\sum_{x\in \bar{x_{k}}}\sum_{y\in \bar{x_{k}}}w_{H}\left(x-y\right)P\left(x\right)P\left(y\right)+\sum_{x\in \bar{x_{k}}}P\left(x\right)\left(1-P_{k}\right)\right)\\ \leq & \sum_{k=1}^{q}\left(P_{k}^{2}\cdot 2\left(1-\frac{1}{\left|J\right|}\right)+P_{k}\left(1-P_{k}\right)\right)\\ \leq & \sum_{k=1}^{q}P_{k}\cdot 2\left(1-\frac{1}{\left|J\right|}\right)=2\left(1-\frac{1}{\left|J\right|}\right)=\eta , \end{array}$$

where we used that $2\left(1-\frac{1}{\left|J\right|}\right)\geq 1$ since |J|≥2 in the last inequality. □

To complete the Plotkin bound for the overweight, we now follow the steps in [10]. Using Proposition 5, we obtain the following result:

**Proposition** **6.** *Let $C\subseteq R^{n}$ be a (not necessarily linear) code of minimum overweight distance d. Then,*

$$|C|(|C|-1)d\leq \sum_{x\in C}\sum_{y\in C}D\left(x,y\right)\leq {\left|C\right|}^{2}n\;\eta .$$

**Proof.**  The first inequality follows since the distance between all distinct pairs of *C* is at least *d*.

For the second inequality, let $p_{i}:R^{n}\to R$ be the projection onto the *i*th coordinate. Note that

$$P_{i}\left(z\right):=\frac{|p_{i}^{-1}\left(z\right)\cap C|}{\left|C\right|}$$

defines a probability distribution on *R* for all i∈{1,…,n}. Using Proposition 5, we obtain that

$$\begin{array}{cc} \sum_{x\in C}\sum_{y\in C}D\left(x,y\right)\; & =\sum_{i=1}^{n}\sum_{x\in C}\sum_{y\in C}W\left(x_{i}-y_{i}\right)\\ \; & =\sum_{i=1}^{n}\sum_{r\in R}\sum_{s\in R}W\left(r-s\right)P_{i}\left(r\right)P_{i}\left(s\right){\left|C\right|}^{2}\\ \; & \leq {\left|C\right|}^{2}\sum_{i=1}^{n}\eta ={\left|C\right|}^{2}n\eta . \end{array}$$

Thus, we obtain the claim. □

From this inequality, we obtain a Plotkin bound for the overweight distance. As before, *R* is a local ring with non-zero maximal ideal *J* and $\eta =2\left(1-\frac{1}{\left|J\right|}\right)$.

**Theorem** **3** (Plotkin bound for the overweight distance). *Let $C\subseteq R^{n}$ be a (not necessarily linear) code of minimum overweight distance d=D(C) and assume that d>nη. Then,*

$$\left|C\right|\leq \frac{d}{d-n\eta }.$$

**Proof.**  We divide both sides of the inequality in Proposition 6 by |C| to obtain that

|C|(d−nη)≤d.

The result then follows from the assumption that d−nη>0. □

By rearranging the same inequality, we also obtain the following version of the Plotkin bound, which does not require the assumption that d>nη.

**Corollary** **4.** *Let $C\subseteq R^{n}$ be a (not necessarily linear) code with ∣C∣≥2 and let d=D(C). Then,*

$$d\leq \frac{|C|n\eta }{|C|-1}.$$

**Proof.**  We obtain this by dividing both sides of the inequality in Proposition 6 by |C|(|C|−1), which is non-zero by assumption. □

**Remark** **2.** *Note that W is a homogeneous weight on $Z_{4}$, and thus our bound coincides with the bound from [10] for the homogeneous weight on $Z_{4}$.*

**Example** **5.** *If we consider codes over $Z_{9}$ and fix |C|=9, n=3. We obtain that d≤9/2 and hence by the integrality that d≤4. The linear code*

C=〈(1,1,3)〉

*attains this bound.*

## 5. A Johnson Bound for the Homogeneous Weight

Another interesting bound is the Johnson bound due to its relation with list-decodability. In the classical form, the Johnson bound gives an upper bound on the largest size $A_{q}\left(n,d,w\right)$ of a constant-weight *w* code over $F_{q}$ of length *n* and minimum Hamming distance *d*. However, for the list-decodability of a code, we are interested in codes having codewords of weight *at mostw*. In fact, if the largest size of such a code $A_{q}^{\prime }\left(n,d,w\right)$ is small, e.g., at most a constant *L*, then every ball of radius *w* contains at most *L* codewords and hence one can decode a list of a size at most *L*. In more detail, the Johnson bound for list-decodability in the Hamming metric states that, if

$$\frac{w}{n}<\left(1-\frac{1}{q}\right)\left(1-\sqrt{1-\frac{q}{q-1}\delta }\right)=J_{q}\left(\delta \right),$$

where δ denotes the relative minimum distance, then $A_{q}^{\prime }\left(n,d,w\right)\leq n\left(d-1\right).$

This famous bound is still missing for the well-studied homogeneous weight, which is, like the overweight, a generalization of the Lee weight over $Z_{4}$. In this section, we prove a Johnson bound for the homogeneous weight from Definition 6, denoted by wt and let γ be its average weight on *R*. By abuse of notation, we denote with wt also the extension of wt to $R^{n}$, that is,

$$wt\left(x\right)=\sum_{i=1}^{n}wt\left(x_{i}\right).$$

Note that wt does not necessarily satisfy the triangle inequality. In [7] (Theorem 2), it is shown that the homogeneous weight on $Z_{m}$ satisfies the triangle inequality if and only if *m* is not divisible by 6.

We define the ball of radius *r* with respect to a homogeneous weight wt to be the set of all elements having distance less than or equal to *r*.

**Definition** **10.** *Let $y\in R^{n}$ and $r\in R_{\geq 0}$. The ball $B_{r,wt}\left(y\right)$ of radius r centered in y is defined as*

$$B_{r,wt}\left(y\right):=\left\{x\in R^{n}\;|\;wt\left(x-y\right)\leq r\right\}.$$

Our aim is to provide a Johnson bound for the homogeneous weight over Frobenius rings. Thus, we begin by defining list-decodability.

**Definition** **11.** *Let R be a finite ring. Given $\rho \in R_{\geq 0}$, a code $C\subseteq R^{n}$ is called (ρ,L) list-decodable (with respect to wt) if, for every $y\in R^{n}$, it holds that*

$$|B_{\rho n,wt}\left(y\right)\cap C|\leq L.$$

Over Frobenius rings, the following result holds, which will play an important role in the proof of the Johnson bound.

**Proposition** **7** ([10] (Corollary 3.3)). *Let R be a Frobenius ring, $C\subseteq R^{n}$ a (not necessarily linear) code of minimum distance d and ω=max{wt(c)|c∈C}. If ω≤γn, then*

$$|C|(|C|-1)d\leq \sum_{x,y\in C}wt\left(x-y\right)\leq 2{\left|C\right|}^{2}\omega -\frac{{\left|C\right|}^{2}\omega^{2}}{\gamma n}.$$

With this, we obtain an analogue of the Johnson bound for the homogeneous weight.

**Theorem** **4.** *Let R be a Frobenius ring and $C\subseteq R^{n}$ be a (not necessarily linear) code of minimum distance d. Assume that ρ≤γ. Then, it holds that C is (ρ,dγn) list-decodable if one of the following conditions is satisfied:*

*(i)*  *We have that γn(d−γn)≥1.*
*(ii)*  *It holds that $\rho \leq \gamma -\sqrt{\left(\gamma -\frac{d}{n}\right)\gamma +\frac{1}{n^{2}}}$.*

**Proof.**  Assume that e≤ρn and let $y\in R^{n}$. We have to show that, under the given conditions, $|B_{e,wt}\left(y\right)\cap C|\leq d\gamma n$.

Note first that we may assume that y=0; otherwise, simply consider the translate

$$C^{\prime }=\left\{c-y\;|\;c\in C\right\}.$$

Assume that $x_{1},\ldots ,x_{N}$ are in $B_{e,wt}\left(0\right)\cap C.$ We have that $wt(x_{i}-x_{j})\geq d$ for i≠j, thus using Proposition 7 and wt(x−y)=wt(y−x), we obtain that

$$N\left(N-1\right)d\leq 2\sum_{i<j}wt\left(x_{i}-x_{j}\right)\leq 2N^{2}e-\frac{N^{2}e^{2}}{\gamma n}.$$

Hence, it follows that

$$N(d\gamma n-2e\gamma n+e^{2})\leq d\gamma n.$$

It holds that

$$d\gamma n-2e\gamma n+e^{2}={\left(n\gamma -e\right)}^{2}-n\gamma \left(n\gamma -d\right).$$

If we assume that nγ(nγ−d)≤−1, then we clearly have

$${\left(n\gamma -e\right)}^{2}-n\gamma \left(n\gamma -d\right)\geq 1.$$

If this is not the case, we see that $\sqrt{\left(\gamma -\frac{d}{n}\right)\gamma +\frac{1}{n^{2}}}$ is well-defined. Thus, if

$$\frac{e}{n}\leq \gamma -\sqrt{\left(\gamma -\frac{d}{n}\right)\gamma +\frac{1}{n^{2}}},$$

then

$$n\gamma -e\geq \sqrt{(n\gamma -d)n\gamma +1},$$

and hence

$${\left(n\gamma -e\right)}^{2}-n\gamma \left(n\gamma -d\right)\geq 1.$$

It follows that N≤dγn. □

**Remark** **3.** *Note that the second condition already forces ρ≤γ.*

**Example** **6.** *As an easy example, we can consider the code $C=\langle \left(1,1\right),\left(4,0\right)\rangle \subset Z_{8}^{2}$ of minimum homogeneous distance 2 and γ=1. The second condition of Theorem 4 is clearly satisfied by choosing ρ=1/2 since*

$$\gamma -\sqrt{\left(\gamma -\frac{d}{n}\right)\gamma +\frac{1}{n^{2}}}=\frac{1}{2},$$

*implying that the code is (1/2,4) list decodable. For example, when setting y=(1,2), we see that*

$$B_{1,wt}\left(y\right)\cap C=\left\{\left(1,1\right),\left(2,2\right),\left(1,5\right),\left(6,2\right)\right\},$$

*so the bound is attained.*

## 6. Open Problems

We conclude this paper with some interesting open questions for the newly defined overweight that we have encountered.

**Problem** **1.** *Classify the codes that attain the bounds derived in this paper.*

**Problem** **2.** *Give a Griesmer bound, an Elias-Bassalygo and a Johnson bound for the overweight.*

Proving an analogue of a Griesmer, Elias-Bassalygo and Johnson bound poses a difficult challenge over rings and in particular for the overweight, due to the necessity of an effective upper bound on the sum of the distances.

## Figures and Tables

**Table 1:** Comparison of weights in $Z_{6}$.

	$w_{H}$	wt	$w_{L}$	$w_{K}$	*W*
0	0	0	0	0	0
1	1	1/2	1	1	1
2	1	3/2	2	2	2
3	1	2	3	1	2
4	1	3/2	2	2	2
5	1	1/2	1	1	1

**Table 2:** Comparison of weights in $Z_{2}\times Z_{2}$.

	$w_{H}$	wt	$w_{B}$	*W*
(0,0)	0	0	0	0
(0,1)	1	2	2	2
(1,0)	1	2	2	2
(1,1)	2	0	1	1

## Data Availability

Not applicable.
